# Interdisciplinary collaboration and clinical management for Norwegian preschool children who stutter: ‘Who, what, when, and where?’

**DOI:** 10.1080/02813432.2025.2531965

**Published:** 2025-07-14

**Authors:** Melanie Kirmess, Karianne Berg, Elisabeth Holm Hansen, Karoline Hoff, Hilde Hofslundsengen, Linn Stokke Guttormsen

**Affiliations:** ^a^ Department of Special Needs Education, University of Oslo, Oslo, Norway; ^b^ Faculty of Education and Arts, Nord University, Levanger, Norway; ^c^ Faculty of Health and Social Sciences, University of South- Eastern Norway, Porsgrunn, Norway; ^d^ Statped, Division of Speech/Language, Oslo, Norway; ^e^ Faculty of Education, Arts and Sports, Western Norway University of Applied Sciences, Sogndal, Norway

**Keywords:** Childhood stuttering, early childhood professionals, stuttering management, early intervention, interdisciplinary collaboration

## Abstract

**Purpose:**

Childhood stuttering may have long-lasting effects on a child’s linguistic and psychosocial development. Early interventions have shown promising results, however, clarity in professional roles and collaboration with parents is warranted to ensure equal and best practice. This study investigated early childhood professionals’ and parents’ experience with interdisciplinary collaboration around preschool children who stutter.

**Method:**

Three focus groups and eight individual digital interviews were conducted with a total of 18 participants: general practitioners (*n* = 2), public health nurses (*n* = 3), speech-language pathologists (*n* = 4), preschool teachers (*n* = 4) and parents (*n* = 5).

**Results:**

Qualitative content analysis resulted in three themes: collaboration routines, competencies in early intervention, and organization of services. Our informants described dual collaborations among the professionals, typically between speech-language pathologists and preschool teachers, especially if the children did not have any other difficulty than stuttering. The professionals had different views on the wait-and-see approach. Both parents and professionals indicated that the system around a child who stutter could be person-dependent in referral and management. Some of the parents experienced that they had to actively seek information themselves to get what their child needed.

**Conclusion:**

This potential inequality of services for preschool children who stutter implies a need for a systematic structure and increased professional knowledge in the healthcare and educational setting.

## Introduction

Stuttering, a multifactorial neurodevelopmental disorder [52] disrupts the fluency of speech, involving repetitions (‘mu-mu-mummy’), prolongations (‘mmmmummy’) and/or blocks (‘….mummy’). Up to 11% of all preschool-aged children experience stuttering [41] involving emotional reactions (e.g. frustration, peer teasing) or behavioral changes (e.g. withdrawal) [27,20]. Even though many children recover naturally from stuttering by the age of 7 [25,44], stuttering that persists into adulthood can negatively impact their social, emotional, and occupational well-being [22,31]. Research indicates that early intervention can effectively mitigate stuttering [7], with immediate treatment recommended if a child shows distress or if parental concern is high [3]. Nevertheless, a simplistic attribution of causality between early intervention and stuttering recovery may not adequately capture the disorder’s complexity, as no intervention has thus far succeeded in entirely eliminating stuttering in all of the treated children (see for example, 10]. Hence, early intervention should also encompass inclusion and equality, emphasizing acceptance and diversity [50]. Achieving this may involve adjusting the child’s communication environment and altering attitudes [53], as well as improving peer relationships and quality of life through initiatives like ‘Camp Dream. Speak. Live’ [9].

Individual and systemic early intervention approaches may involve several professionals in addition to the parents. However, there is limited knowledge of early childhood professionals’ collaboration around a child who stutters or the professional support parents seek. Therefore, this study explores the collaboration between parents and professionals in supporting young children who stutter.

### State of the art regarding collaboration management

Interdisciplinary collaboration can be defined as ‘two or more healthcare professionals who have specific roles, perform interdependent tasks, and share a common goal’ [14,23, p. 71). Despite terminology variations, the literature agrees upon key elements such as communication, trust, respect, patient/person-centeredness, mutual acquaintanceship, task characteristics, power, formalization, and professional role clarification [23,39]. The child and their parents should be acknowledged as stakeholders of the current situation and be involved early for common goal setting [15].

Stuttering may evoke parents worries and frustration in relation to their child’s social-emotional well-being and future [16,20,27]. Therefore, parents of children who stutter require knowledge and guidance on how to manage this condition and the related environmental reactions [40]; including information on stuttering, referrals to speech-language therapy (SLT), and guidance on how to support their child [4]. Depending on the cultural context, parents contact different professionals for advice on stuttering management [35].

#### Starting collaboration: identification and referral

In Norway, SLT requires a healthcare or educational system referral, typically presented by parents in collaboration with preschool teachers, public health nurses, or general practitioners (GP) [24]. If the municipality does not employ a speech-language pathologist (SLP), referral to private SLT is provided by GPs [34,47).

Norwegian public health nurses and GPs are regularly involved in the healthcare of all children, with public health centers offering a standard consultations between ages two and four years to identify delayed development or disorders, including stuttering [36]. Over 97% of families attend these consultations [45]. Public health nurses can support identification of stuttering by listening to the child’s speech, and parents can express concerns and ask for information about stuttering [21].

GPs can contribute to early identification of stuttering and support parents in coping with reactions such as anxiety and frustration [43]. Two American studies investigated the referral practice of GPs, discovering that they (a) often recommend a wait-and-see approach to parents when they lack a specific screening tool [54], and (b) are more likely to identify and refer children who stutter if they observe negative communication attitudes and/or stuttering-like disfluencies during the visits [51].

Approximately 93% of all preschool children (aged 1–5 years) attend preschool daily in Norway [46]. The Norwegian preschool teacher’s professional role includes the identification of children at risk for language and communication disorders [33]. However, not all preschool teachers have research-based knowledge of stuttering to be able to identify themselves [24]. Instead, they can rely on assumptions, such as the incorrect belief that stuttering is caused by psychological issues [30]. The Norwegian preschool teachers in Kefalianos et al. [24] study emphasized children’s reactions to stuttering, its severity, and parental concern when considering further referral. This aligns with findings from other studies showing that parents are highly accurate in identifying stuttering in their children [11,48].

#### Management of collaboration: assessment and treatment

Stuttering assessment includes three main perspectives of information; the person who stutters, the family surrounding the person, and the clinician [8]. In addition, teachers can have relevant information regarding the child′s stuttering in preschool and other’s reaction to the stuttering. Therefore, collaboration is essential in assessment. Previous studies highlighted the importance of an interdisciplinary team [6] and collaboration [12], including a second opinion and validation of stuttering management discussions between SLPs. Further, to manage individual needs, SLPs need experience with evidence-based stuttering treatments [5,49].

Parents play a key role in the treatment of preschool children who stutter, because most treatment programs involve SLPs guiding parents to provide treatment for their preschool children at home [e.g. 1,13,26,38]. Parents who are involved in their child’s stuttering treatment become more knowledgeable, less worried and more confident in managing [32].

### Current study

The Norwegian legislation expects collaboration among early childhood professionals, for example, when a GP refers a child to private SLT [47], or when a preschool teacher refers a child to SLT in the municipality [33]. Despite the important role of GPs as gatekeepers for referral to stuttering therapy through the healthcare system, no study has investigated their practice with preschool children who stutter in Norway. Given that in Norway, SLT is often provided in preschool settings [18], it is crucial to clarify the roles of parents and early childhood professionals to ensure the effectiveness of the referral and early intervention processes. Therefore, this study investigates collaboration practices for preschool children who stutter to a) enhance our understanding of barriers and possibilities from the perspectives of parents and early childhood professionals in Norway, and b) to inform practice in early childhood education and care. We explored the following research questions:

1. What needs, expectations and experiences of collaboration do parents and early childhood professionals have around preschool children who stutter?
2. What do parents and early childhood professionals express as the ideal collaboration for preschool children who stutter?

## Method

This is a follow-up study of Norwegian early childhood professionals’ practices regarding preschool children who stutter [24] and part of the Effective Stuttering Treatment project. This study aimed to acquire in-depth information and experiences through focus group interview with early childhood professionals and individual interviews with parents. The project was ethically approved by the Norwegian Centre for Research Data, project number 144522. All participants provided written informed consent.

### Research team and reflexivity

#### Personal characteristics

The female interdisciplinary team consisted of five experienced SLPs s (MK, KB, KH, HH, LSG) and a certified public health nurse (EHH) with research interest in preschool stuttering. KH and LSG worked daily with stuttering treatment. KB and KH have previous teacher education, and HH preschool teacher education. All authors hold a Ph.D.-degree from diverse areas, except for KH, who is a senior stuttering expert representing the clinical field. Data analysis was primary conducted by KB, EHH, MK, all with experience in interview methodology. None of the authors stutter themselves or are parents to children who stutter.

#### Methodological reflexivity

We have strived to ensure a high level of transparency in our descriptions of the research process, encompassing methodological choices and data analysis procedures. Being a group of researchers, we have also been able to address possible personal biases through discussions and consensus decisions. All major decisions have been made collaboratively.

#### Relationship with participants

Since professional networks were involved in recruitment, some of the authors had previous knowledge about possible professional or parent participants. However, interviews were conducted by authors without previous relationship to the participants. MK (moderator) and EHH (secretary) conducted the interviews with the professionals due to their diverse education and healthcare background to reduce a possible bias of inside perspectives from SLT only. Parent-interviews were conducted by HH (interviewee) and KH (secretary) related to their prior experience with parent involvement. In some of these interviews, sharing of the personal histories evoked despair, sadness and anger. As parents, the interviewers recognized and acknowledged these emotions during the interview, despite being unable to offer practical help. The first meeting with the participants were during the interviews.

### Study design

To extract a broader narrative about preschool stuttering collaboration, we planned focus group interviews [29]. The transcribed records were analysed based on Graneheim and Lundman′s (2004) qualitative content analysis.

### Participant selection

Inclusion criteria were early childhood professionals experienced in managing children who stutter, and parents of children who stutter or have stuttered during the last five years. Recruitment first targeted SLPs, public health nurses and preschool teachers consented to follow-up studies in a previous survey [24]. Only two participants were recruited through this strategy with COVID-19 workloads being the main reason for refraining from participating. The authors’ professional networks were used to recruit the remaining participants (including GPs and parents as well) directly through email or phone, sharing written and/or oral information about the study and asking receivers to respond if interested. The final sample included 18 participants.

#### Setting

Parental interviews were conducted separately from the professional interviews, because we wanted the parents to be able to share their personal experiences on a peer level with other parents. Due to the COVID-19 pandemic issues over a longer period, we only were able to conduct three focus group interviews: two for professionals (*n* = 4 and *n* = 4) and one for parents (*n* = 2). We chose to supplement data collection with individual interviews so that all recruited participants could participate. We conducted five individual interviews with professionals and three with parents.

Participants in this study included four preschool teachers, three public health nurses, two GPs, four SLPs and five parents. Three of the professionals were male. All parents were by coincidence highly educated females. Both parents and professionals were recruited from the whole country, representing both urban and rural areas. None of the participants were connected to the same cases. The professionals’ working experience varied from recently graduated to more than 20 years within a profession, and participants worked in both public and private sectors. Experience with children who stutter ranged from meeting one child to working with them daily. Further, none of the participating SLPs described themselves as stuttering specialist or worked only within the field of stuttering. However, all participants expressed a specific interest in the field of stuttering and many also took the opportunity to gain more information about stuttering by participating in the interviews.

### Data collection

A semi-structured interview guide was created to broadly address collaboration and stuttering, ensuring diverse discussion topics. The interview guides within professional and parent groups were similar across interview types (see Supplementary material for the interview guides). All 11 interviews were conducted digitally using Zoom between September 2021 and January 2022. The interviews lasted between 35 and 75 min, on average an hour. The interviews were recorded and saved on the University of Oslo mandatory secure server for identifiable data.

Two research assistants (SLP students) transcribed the video recordings verbatim with the authors validating the transcriptions with the original data.

### Data analysis

The qualitative content analysis [17] included all authors in different phases. First, everybody read through one of the focus group transcripts to agree upon which segments of the text were relevant to the research question. While reading through this first transcript, all authors marked meaning units relevant to the research questions. The meaning units were discussed and data from the meaning units were condensed into preliminary codes. This work formed the basis for codes used in the extended analysis of the entire material, conducted by MK, KB and EHH. To strengthen the common understanding of the analysis process, two interviews were first coded individually by the three authors and then discussed. The results from this round of coding showed a high incidence of code similarity, indicating a common understanding of the phenomenon, but still providing flexibility to add new codes. The remaining interviews were divided among these three authors and coded individually. The NVivo-software [28] was used to ensure a common structure and suitable for sharing codes as there were many people involved. Finally, all authors grouped codes into categories and developed overarching themes and sub-themes that captured the essence of clusters of codes (categories) based on the research questions. The discussion led to the final themes where we sought to secure deeper insight into the data. A few themes that did not fit the data (e.g. politics) were excluded, and additional ones evolved (e.g. low-threshold services). Table 1 provides an example of the analysis process. Table 2 lists all final themes and sub-themes.

**Table 1.** Examples of the coding strategy.

Quotation	Code	Sub-theme	Theme
*We wait. This wait-and-see attitude on the one hand, while we on the other hand are pushed to early intervention that it is important to get started.*	Early intervention	Different views on the wait and see approach	Competencies in early intervention
*Concerning stuttering or other challenges I have questions about, it would have been good to have a dialogue with the speech-language pathologist, for example.*	Collaboration	Collaboration in the identification and referral phases	Collaboration routines

**Table 2.** Overview of research questions, themes, and Sub-themes.

*RQ 1) What needs, expectations and experiences of collaboration do parents and early childhood professionals have around preschool children who stutter?*		
Theme 1	Collaboration routines	*Collaboration in the identification and referral phases* *Collaboration in intervention management and feedback*
Theme 2	Competencies in early intervention	*Access to speech-language pathology services* *Different views on the wait-and-see approach* *Knowledge and competence depend on the individual and profession*
*RQ 2) What do parents and early childhood professionals express as the ideal collaboration around preschool children who stutter?*		
Theme 3	Organization of services	*System for referrals* *More competence from referral to stuttering treatment*

## Results

The analysis resulted in three main themes: collaboration routines, competencies in early intervention, and organization of services and seven sub-themes as presented in Table 2.

### Collaboration routines

In general, collaboration was most often described as happening between two professional groups (e.g. teachers and SLPs) or between parents and one profession, but seldom in extended interdisciplinary settings.

I have no experience with interdisciplinary collaboration meetings when it comes to stuttering. These are rather used in more complex cases. (Public health nurse)

The need for interdisciplinary meetings and collaboration around a child was only expressed if they had complex developmental disorders but not for stuttering alone.

Despite this, all participants expressed strong positive engagement towards collaboration concerning preschool children who stutter. The professionals and parents addressed the concept of collaboration at several levels and in different periods in the management of each case when a child stutters, leading to two sub-themes: collaboration in the identification and referral phases, and collaboration in intervention management and feedback.

#### Collaboration in the identification and referral phase

In cases of concern about stuttering, the professionals described different experiences with collaboration depending on whom the parents contacted first with their concern, which professionals were available in the individual municipality and the knowledge and competence possessed by the individual professional. The parents, preschool teachers and public health nurses were most likely the first to raise concern regarding stuttering in a child. If the public health nurses raised concerns, they would contact the preschool that the child attended, following the parents’ consent. None of the public health nurses had experienced parents who did not want them to contact the preschool. However, if the preschool teacher raised the concern, it was rather unlikely that the local public health nurses were involved. Further, several preschool teachers described that their referral to a SLP was more likely to be taken seriously at the system level if it was supported by observations from healthcarers.

As soon as there is a public health nurse or a GP included, it gets a more serious character. (Preschool teacher)

GPs’ involvement seemed limited to cases which required referral to a private SLP. This did not necessarily require meeting the child in person; both parents and GPs reported referrals based on parents’ requests alone. One of the GPs questioned their need in referrals.

[When the GP is informed beforehand] … collaboration is experienced as more meaningful, and then the GP is not only a secretary, but actually gets the opportunity to contribute. (GP)

Both GPs described their knowledge required to create better services for a child who stutters as limited, even if they had been involved early in the process.

#### Collaboration in intervention management and feedback

Another aspect of collaboration addresses communication between different professional groups and between professionals and parents regarding intervention management. Good or effective communication was described as easy access to information (e.g. after referrals or assessments), being respected and heard when concerned about a child’s possible stuttering, and being included in intervention management.

Concerns regarding collaboration with SLP were raised by several of the other participants. Both parents and preschool teachers described situations in which neither party was involved in the interventions as they had expected and wished to be. The preschool teachers expressed a specific need for supervision, involvement, and feedback from SLPs since they engage with the child daily, whereas the SLP has limited visits.

Except from the one supervision, I feel there was little collaboration around this child who stutters. (Preschool teacher)

The importance of good communication with the SLP was also expressed by the parents, whereas the lack could result in negative feelings and frustration.

[…] not have a wait-and-see focus, but that you can call a SLP or GPs to discuss your concerns. (Parent)

The SLP was very explicit that some people stutter, that we would evaluate our situation, and that the stuttering may not disappear. This was hard to hear, but fine in one way, too. (Parent)

The public health nurses also described the lack of systematic feedback from the SLP.

Routinely, we do not get any feedback other than that the case is accepted. Sometimes, it happens that the SLP wants to discuss or talk about actions we take, but usually, I will get a notice from the parents in these cases. (Public health nurse)

However, the SLPs expressed a desire to involve the others.

I am satisfied when I have met the child, listened to the observations from the parents, and interacted with the preschool teacher. (SLP)

### Competencies in early intervention

In all interviews the participants talked about early intervention, highlighting three sub-themes: access to SLP services, different views on the wait-and-see approach, and knowledge and competence depending on the individual and profession.

#### Access to speech-language pathology services

All participants described the following three main challenges concerning access to SLT services at the system level: (1) lack of SLPs in the municipality; (2) the SLPs’ workload (e.g. long waiting lists, limits for provided treatment hours) and (3) access to SLPs with competency in stuttering treatment.

It takes many years to establish this competence in the municipality. There are just not enough SLPs. (Parent)

Even with a granted right to SLT services from the municipality, this did not necessarily imply an offer in line with stuttering intervention guidelines as experienced by the parents (e.g. the number of hours prescribed).

The assignment/obligation we received in the preschool was one hour every sixth week. […] This is nothing, and you do not get any continuity with that. (Parent)

In addition to the treatment management itself, the parents also expressed a need for parental support.

Both parents and professionals talked about experiences showing how easy access to SLT services could be organized and delivered. Easy access was described as a service in which the child who may stutter got access to a SLP within a very short timeframe after the parents and/or the professional had expressed their concerns.

I am an admirer of that approach; to look into a case as soon as possible, to evaluate and then rather to retract if there is no need. (GP)

Only one parent described a positive, easy access experience.

A SLP from the easy access group in our community visited the preschool and evaluated our child and [based on the level of stuttering and second behaviors] decided to start intervention immediately. In parallel, the SLP referred further to the Educational-Psychological Service. The process took only 3 weeks altogether. (Parent)

However, the more common scenario described difficulties in services access and a mismatch in expectations and delivery.

#### Different views on the wait-and-see approach

Despite the strong early intervention focus, participants (especially parents) met the ‘wait-and-see’ strategy. This strategy was suggested to the parents mainly by professionals other than SLPs. However, both parents and preschool teachers questioned this advice.

We wait. This wait-and-see attitude on the one hand, while we on the other hand are pushed to early intervention; that it is important to get started. I feel we got drawn into two different directions. (Preschool teacher)

The wait-and-see strategy was therefore often perceived as a negative or passive approach across parent and professionals. Furthermore, professional conflicts could arise when different requirements or considerations contravened. Some of the participants experienced not being taken seriously about their concerns for the child’s stuttering by being asked to wait and see. However, the wait-and-see approach could also be a reasonable strategy if it was followed by actively survey the child’s development with certain indicators for further interaction.

We can do the wait-and-see, but then we need to know what we are waiting for and what we should look for. (SLP)

A good structure at the different levels is necessary for a good collaboration. […] At a certain point, it may be more necessary with active SLT at that time, [in other situations]it may be supervision of caregivers, including parents and preschool teachers, possibly supported by further assessment along the road. (GP)

#### Knowledge and competence depend on the individual and profession

The types of information and advice given to parents whose children stuttered depended largely on whom they met. Some parents just had to hope for the best, not knowing how it would end, implying certain resignation. Further, parents described that the assignment of a SLP did not necessarily mean that this person had relevant knowledge within stuttering management or was a specialist in stuttering treatment.

I expect that the Educational Psychological Service and the SLPs are updated on what is going on […] Some of the SLPs that were educated 30 years ago, how little they have read of newer research. (Parent)

It felt good to meet a professional [SLP] who knew what he/she was doing, and who at the same time balanced the fact that the stuttering might continue throughout life. (Parent)

The preschool teachers and public health nurses described that their knowledge and competence regarding stuttering were a result of their personal interest and individual knowledge acquisition. They highlighted the lack of guidelines for stuttering management and relevant information on which advice they could provide to parents.

We do not have in-depth [stuttering] competence; we have rather competence on a broader level and practical experience. (Preschool teacher)

My professional role is to uncover, I am supposed to find the problems and challenges, and then I am supposed to help the family further. (Public health nurse)

If they could prepare a procedure, maybe it would be easier to have focus on it [stuttering]. Easily accessible web pages. […] On a quality secured webpage, for example, the national guidelines [for public health nurses] from the Directory of Health. (Public health nurse)

The parents also emphasized what was perceived as a need for them to actively seek information about stuttering, thereby increasing their own competence. Several parents indicated that this search for information and knowledge was driven by their impatience with a slow-running system, or because they questioned whether the service their child received was the best and evidence-based.

It should not be necessary that you have to do so much research yourself as a parent. (Parent)

Considering this, both parents and professionals were concerned that resourceful parents recieved more adequate help, to more likely have capacity to search for information and thereby demand what they found to be the best intervention for their child.

So, you get this unbalance in who has the resources. I do not mean the economical ones, but human resources. That means, parents that have the capacity to follow-up, to nag at people, to take responsibility, those that don’t accept a no for a no, those who push forward. They get the help. (Parent)

The parents suggested the need for easily accessible information about stuttering and its management for everybody. Contact with peers in the same situation was useful for additional information and support.

### Organization of services

All participants addressed the organization of services for children who stutter. These experiences were conceptualized into the sub-themes: system for referrals and, more competence from referral to stuttering treatment.

#### System for referrals

All professionals discussed the lack of a unified system for referrals to SLPs, included where to send referrals and the following referral review process. One participant described referrals that had been rejected without a SLP having viewed or processed them. Therefore, the participants suggested that the reception team for referrals must include access to a SLP. As described by the participants’, attempts to question what was perceived as a non-functional system in their municipality was met with a ‘This is how we have chosen to organize it’ attitude, and no changes appeared. Both professionals and parents mentioned ‘person-dependent’ several times as a keyword regarding the referral system. This was described as each individual and each municipal having their own way of handling these matters.

To have something easily accessible, a procedure or the like describing what does the healthcare station do in detail at this point. It should be independent of which municipality you work in. (Public health nurse)

*‘Who, what* and *when’* was explicitly described by one of the GPs as the important questions related to the process of referring and initiating SLT for preschool children. In addition, many participants described the systemic case processing as highly time-consuming.

We did expect more persons working at the Educational Psychological Services and hence, less long waiting lists. (Parent)

[The authorities] agreed that our child did not get enough support, but they did not do anything about it. (Parent)

On the opposite, a good referral example included the co-location of several services (public health nurse and SLP), resulting in faster management of stuttering and daily meeting points to discuss smaller concerns.

#### More competence in referral to stuttering treatment

The competence required to identify stuttering and thereby breaking ground for a referral was also discussed. A GP suggested that a preschool professional skilled in speech and language development should handle referrals to SLPs, rather than GPs.

One can question if it is necessary to have a doctor to submit a referral. Because the document you want and need for a referral, is actually based on observations from the preschool, school and parents. (GP)

The parents also asked for a systematic structure for stuttering management, comparable to healthcare packages, as they exist for cancer care in Norway.

A patient pathway for different disorders, where you get information and what you need to do is desired. (Parent)

A healthcare package should contain information about the stuttering disorder and the management system from referral to treatment. In line, preschool teachers and public health nurses requested tools and meeting places parental advice.

The ideal would be, that if it happens, you find a structure for it: ‘first you do that, and then call them’ (Preschool teacher)

I wished we had regular meetings with the SLP in our municipality… Then we could discuss with them. (Public health nurse)

Such meeting places could support discussions and offer answers concerning stuttering thereby improving their own competence.

## Discussion

This study explored collaboration practices for preschool children who stutter to enhance the understanding of barriers and possibilities of accessing best practice from the perspectives of parents and early childhood professionals in Norway. Overall, systemic collaboration lacks structure from referral to management of preschool stuttering. Our findings revealed a large variation in routines for referrals and access to SLP services and thereby inequality in what is offered to the children and parents. Formalization and organizational structures are key elements in a good collaboration, and hence, the lack of such increases barriers and restricts the best outcome for the service [23,39]. However, a stronger organizational structure should not overpower tailored individual management services, as children who stutter are a heterogeneous group [15,44].

Our findings describe limited resources for delivery of early intervention which do not align with the requirements for delivering evidence-based stuttering treatment programs [2,7]. One possible reason could be the limited number of available SLPs, a challenge that is also well known from an international perspective [12]. Further, local organizational factors restrain the available amount of therapy time for public SLPs.

### Roles and competence in collaboration among childhood professionals

Our results support that early identification of stuttering by early childhood professionals based on parental concerns is a key element in referrals and access to SLT.

The limited knowledge and competence of various professionals can be a barrier, especially for early referrals. Both preschool teachers and public health nurses previously reported suggesting a ‘wait-and-see’ approach when parents first contact them [24], inconsistent with stuttering recommendations [3,37]. Nevertheless, stuttering severity, parents’ concern, and the children’s age, were recognized as important referral factors, consistent with other studies [49].

The GPs discussed their role in the referral process. Even though pediatricians are more likely to refer children with observable stuttering behaviors or negative attitudes towards communication than previously, a concern was raised for children who do not stutter during the consultation [51]. This concern supports the Norwegian GP’s suggestions that other professionals with more competence in SLT and professionals who are frequently meeting the children were better suited to make referrals.

Preschool teachers require closer collaboration with the SLP to supervise and actively support the management of the child’s stuttering during everyday life in preschools. Preschool teachers can be a valued resource in stuttering management because Norwegian children spend a large part of the day in childcare. Trained preschool teachers could observe how stuttering affects the children and take actions related to the stuttering referral [19]. To the best of our knowledge, no study has yet investigated the role of preschool teachers in managing stuttering.

SLPs competence and evidence-based knowledge of different stuttering approaches are key factors for tailoring and conducting successful stuttering treatments [5]. However, our findings highlight a much more diverse reality with limitations within the competence and confidence of SLPs as experienced by parents and other professionals. The lack of confidence for stuttering treatment hat general SLPs describe can adversely affect the clinical services provided to this group [8]. Therefore, any recommendations for stuttering treatment should address levels of general and specialized competencies to meet the child’s needs in different phases of stuttering management.

### Challenge of early intervention

The optimal early intervention routine describes a scenario in which the child who stutters access services within a very short timeframe after raised concerns. All participants discussed how ‘early’ early intervention should be and what to wait for in a wait-and-see approach. These important questions are regularly discussed in the field of stuttering [27,37]. Considering that stuttering increases the likelihood of poor emotional and social functioning [31], treatment close to stuttering onset is generally recommended. If treatment is delayed, or a wait-and-see approach is advocated, this should be done together with a careful observational approach of both stuttering and its impact on the child [51]. Importantly, early referrals did not necessarily equal early intervention. Even if the municipality dutifully assessed the child early, they may not be able to offer treatment before much later, at a lower dose, or by a SLP lacking relevant stuttering competence. Those findings raise major concerns when it comes to prevention of the possible serious consequences untreated stuttering can lead to.

### Parents’ needs when their child stutters

Children who stutter and their parents are the key stakeholders when they address professionals [15]. The parents were primarily concerned about receiving the best stuttering management for their child consistent with previous findings [4,27,35,40]. Furthermore, they also searched stuttering advice and social and emotional support from a SLP, a finding in line with Nonis et al. [35] and Erickson et al. [12].

Parents received different stuttering advice from different professionals. They therefore argued for the need of easily accessible guidelines for actions to take in stuttering management (e.g. patient pathways or national guidelines for stuttering management).

Both parents and professionals were concerned that parents with better educational or socio-economical resources receive more adequate help, because they have better access to relevant information, and they are more likely to have stamina to fight the system. Safwat and Sheikhany [42] findings support that high-educated parents had more knowledge about stuttering etiology, treatment and prognosis. Coincidentally, or maybe biased by the recruitment process, the parents in our study represented a high educational background and hence, elaborated on their search for knowledge and how that information helped in receiving the stuttering treatment required for their children.

### Limitations of the study

First, even with online interviews, recruitment was challenging within the changing regulations of the COVID-19. We would prefer a higher number of GPs and public health nurses to strengthen the broader perspectives from the healthcare system. Further, participants who agree to participate may have stronger opinions and engagement in the topic than those who did not answer. Some participants specifically expressed aim to gain more knowledge about stuttering management through the study. Therefore, the results must be interpreted cautiously within this context. Second, focus group as a method was primarily chosen to elicit interactive and meta-discussions that are usually limited in traditional interviews. The inclusion of a high proportion of individual interviews may have evoked responses other than that in a group setting. However, with the choice of having limited data due to meeting time limitations or conducting additional individual interviews, we preferred the latter to cover a broader range of experiences and thoughts. Having conducted the focus group interviews before the individual interviews, we were able to bring up discussions from the focus groups into the individual interviews.

## Conclusion

Parents and early childhood professionals embrace collaboration, even though none of the early childhood professionals expressed the need for extended professional meetings if it concerned stuttering alone. However, professionals and parents clearly described the need for effective communication and information transfer across all system levels. The involvement of the parents, including acknowledgements of their needs and feelings throughout the different stages from referral to intervention, is crucial. Furthermore, all participants highlighted the need for easily accessible guidelines for managing preschool stuttering, addressing what to do by whom and when, to secure equal possibilities in stuttering management for all children. To raise awareness and improve knowledge of stuttering among early childhood professionals, SLPs could contribute to the respective education programs to increase accuracy in stuttering identification. Furthermore, there is a need for continuous education offered within evidence-based stuttering treatment to enhance competence among SLPs.

## Supplementary Material

Supplementary material Interview guides.docx

## Data Availability

The anonymized transcripts of the interviews are only available in Norwegian. The datasets generated during and/or analysed during the current study are available from the corresponding author on reasonable request.
