# Supplementary material for: Interdisciplinary collaboration and clinical management for Norwegian preschool children who stutter: ‘Who, what, when, and where?’
Source: Scand J Prim Health Care. 2025 Jul 14;44(1):1–14. doi: 10.1080/02813432.2025.2531965 (PMC12918392; doi:10.1080/02813432.2025.2531965)
Supplement: Supplementary material Interview guides.docx [file IPRI_A_2531965_SM9940.docx]

# Supplementary material – Semi-structured interview guides

## Interview guide for the professionals

- Can you describe your last referral or contact you received concerning a child who stutters? Would you say that it was a typical referral?
- Which factors or elements do you think are important in the referral process? Is there something in the referral process that is important for collaboration? Do you have any examples?
- What is the first thought you have when you think about collaboration? Do you have any experience or examples of collaboration? What do you think why there was or was not a collaboration? Please provide both good and not-so-good examples.
- Given the case in which you were part of a group of professionals around a child who stuttered, which expectations would you have for each other? Exemplify. Who do you actually collaborate with? Are there any reasons why you are not collaborating with profession x, y or z?
- Do you have any examples of how mutual expectations can be more outspoken or explained to each other?
- Which expectations do you have for collaboration with parents?
- If you think about the ideal collaboration, what would that look like? Which prerequisites need to be in place for an ideal collaboration? What are typical barriers to reaching this today?

## Interview guide for parents

- Can you tell us in short how the journey was from you discovered that your child stutter to you got in contact with a speech-language pathologist? Examples?
- What experiences have you had with collaboration around your child who stutters? Whom have you worked with; speech-language pathologist? Teachers? Public health nurses? General practitioners?
- What was good and what could have been better in this collaboration?
- Expectations: what expectations did you have to speech-language pathologists, teachers, public health nurses, and general practitioners related to your child who stuttered? Examples?
- What needs to be present to build a good collaboration? What do you think are the biggest barriers to this today?
- What experience have you had in the transition from preschool to school for your child who stutter?
